# The Use of Amantadine in a Nontraumatic Disorder of Consciousness: A Case Report

**DOI:** 10.7759/cureus.107262

**Published:** 2026-04-17

**Authors:** Mary Hanna, Joe Chen, Diana Salama

**Affiliations:** 1 Family Medicine, Loma Linda University Medical Center, Loma Linda, USA

**Keywords:** amantadine, brain trauma injury, disorders of consciousness, stimulants, stroke

## Abstract

Disorders of consciousness (DoC) are among the most challenging conditions encountered in clinical practice, often complicating both diagnosis and management. Although amantadine has demonstrated benefit in facilitating recovery in patients with traumatic brain injury (TBI)-related DoC, its impact on nontraumatic causes has not been well established. We describe the case of an elderly patient with persistent encephalopathy following an ischemic stroke who experienced marked neurological improvement shortly after initiation of amantadine therapy. This case highlights the potential role of amantadine as a therapeutic option in non-TBI-related disorders of consciousness.

## Introduction

Disorders of consciousness (DoC) encompass a range of conditions from coma to the minimally conscious state. Although non-traumatic causes of DoC, including stroke and metabolic encephalopathy, can be equally debilitating, the majority of current research has focused on treatment strategies for traumatic brain injury-related encephalopathy [1]. Recovery from DoC may improve with the resolution of both primary and secondary neurological insults; however, many patients remain unresponsive despite correction of metabolic abnormalities or appropriate treatment of the underlying cause, including infections [1].

Amantadine, an indirect dopamine agonist and NMDA (N-methyl-D-aspartate) receptor antagonist, has demonstrated benefit in accelerating early recovery in traumatic brain injury (TBI) patients through mechanisms thought to improve arousal and attention via dopaminergic stimulation [1-3]. Although it is FDA-approved for Parkinson’s disease and drug-induced extrapyramidal symptoms, it is used off-label in DoC. Its use in non-TBI patients, such as those with cerebrovascular disease, remains less well defined [2]. This case highlights the value of considering amantadine in selected patients with non-traumatic causes of impaired consciousness despite correction of metabolic abnormalities or infectious causes.

## Case presentation

A 94-year-old woman with a medical history notable for hypothyroidism, Alzheimer’s dementia, complete heart block status post pacemaker placement, recurrent urinary tract infections, and a recent episode of pneumonia presented to the emergency department with decreased oral intake, altered mental status, and acute respiratory failure. According to her family, she had previously been alert, oriented, and socially engaged at baseline.

On presentation, the patient’s Glasgow Coma Scale (GCS) score was 8-9. She required supplemental oxygen and was admitted to the intensive care unit for close monitoring and treatment of sepsis. Initial neuroimaging with computed tomography (CT) of the brain showed no acute abnormalities, including hemorrhage or acute infarcts. Magnetic resonance imaging could not be performed due to the presence of a pacemaker.

The initial workup identified concurrent urinary tract and lower respiratory tract infections, and she was started on intravenous antibiotic therapy. Over the following 72 hours, her vital signs and inflammatory markers improved, with stabilization of her clinical status. 

**Figure 1:**
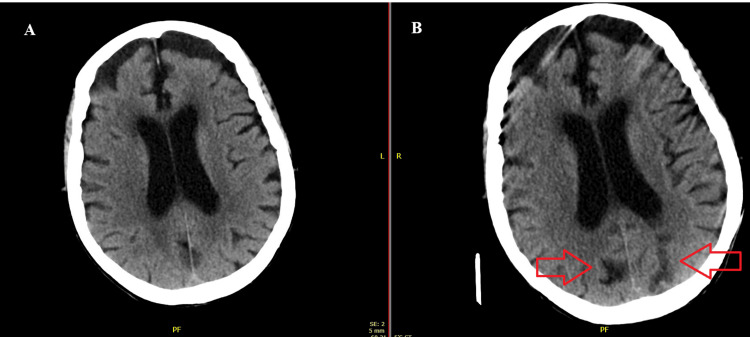
Initial CT head without contrast (A) vs subsequent CT head with and without contrast (B) with new subacute occipital infarct

**Figure 2:**
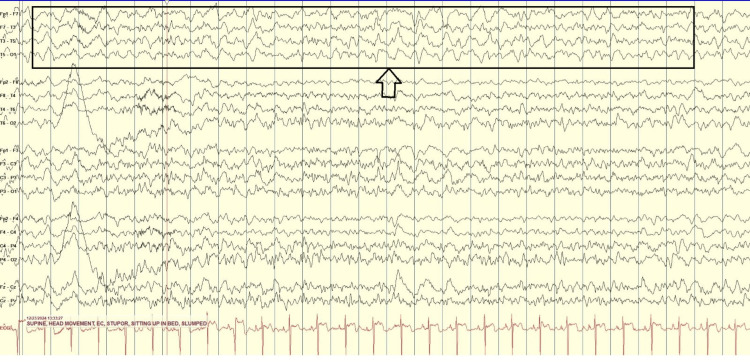
Intermittent focal slowing in the left temporal channels. Diffuse background slowing consistent with diffuse encephalopathic state

Although the infectious process resolved and metabolic abnormalities, including electrolyte, thyroid, and renal function, were corrected, the patient’s mental status showed little to no improvement. Electroencephalography (EEG) revealed generalized slowing consistent with diffuse encephalopathy, without epileptiform discharges, which prompted repeat CT imaging. CT of the head with and without contrast demonstrated multiple subacute infarcts not seen on initial studies, raising concern for a cerebrovascular contribution to her persistent encephalopathy (Figures 1, 2). 

Treatment pathway

She was treated with antibiotics for her infections and continued on levothyroxine for her hypothyroidism for approximately 2.5 weeks; however, her mental status did not improve. She remained significantly somnolent and was unable to be aroused by auditory or tactile stimuli, including a moderate sternal rub.

Due to her persistent altered mental status despite comprehensive management, a multidisciplinary team, including neurology and geriatrics, recommended a trial of amantadine. She was initially started on amantadine 100 mg in the morning, with notable improvement observed the following day, as the patient began opening her eyes to voice, tracking visually, and demonstrating purposeful movements. This prompted an increase in the dose after two days to 100 mg twice daily (at 0900 and 1300 hours) to minimize nighttime stimulation, with close monitoring of her response to the medication.

Outcomes

On the fourth day of amantadine therapy, the patient showed marked neurological improvement. Her GCS improved to 14-15, and she began following commands, engaging in conversation, and tolerating oral intake. Her cognition returned close to her baseline. She was transitioned to oral medications, completed antibiotic therapy, and was discharged to a subacute rehabilitation facility. She recovered well at the rehabilitation facility and remained at her baseline mental status prior to admission. She was subsequently discharged home with home health.

## Discussion

This case shows the potential benefit of amantadine in the treatment of DoC resulting from nontraumatic brain injuries such as stroke-related encephalopathy. The dramatic neurological recovery following initiation of amantadine, after infectious, metabolic, and structural causes had been addressed, supports its role as an adjunct in carefully selected patients.

Amantadine's mechanism of action is not completely clear, though it involves increasing synaptic dopamine availability by increasing dopamine release and blocking dopamine reuptake, particularly in the prefrontal cortex and basal ganglia, which may enhance arousal, motivation, executive function, neuroplasticity, and cortical connectivity [1-3]. It blocks NMDA-type glutamate receptors, which may reduce excitotoxicity and is thought to reduce neuronal damage from overstimulation [3]. The neurobehavioral effects are likely to result from increased neurotransmission within dopamine-dependent pathways, specifically the nigrostriatal, mesolimbic, and frontostriatal circuits, which play key roles in regulating arousal, motivation, and attention [1,3]. These effects are notably observed in patients with diffuse cerebral injury or disrupted neural networks, even in the absence of traumatic brain injury [4-6].

What does the evidence show?

In TBI, Non-ICU

A large, multicenter randomized controlled trial conducted by Giacino et al. in 2012 demonstrated that amantadine (100 mg twice daily) significantly accelerated functional recovery over a four-week period compared with placebo in patients with severe traumatic brain injury-related disorders of consciousness undergoing inpatient rehabilitation [2]. This study represents the most rigorous evidence supporting the use of amantadine as a neurostimulant and provides guidance regarding dosing and the expected timeframe for clinical response, typically within two to four weeks [1,2]. Subsequent reviews and meta-analyses have reinforced these findings and further supported its role in promoting recovery after traumatic brain injury [3,4]. Although the study population consisted of non-ICU patients with traumatic brain injury, its findings have informed the broader application of amantadine in disorders of consciousness [1,2].

Most published studies report observable clinical improvement within two to four weeks of initiating therapy, and expert consensus guidelines recommend reassessment during this interval using standardized neurobehavioral scales such as the Coma Recovery Scale-Revised (CRS-R) to evaluate treatment response [2]. Earlier studies assessing amantadine in TBI often used the Disability Rating Scale (DRS), which represents a higher threshold for functional improvement. More recent literature has increasingly adopted the CRS-R due to its greater sensitivity in detecting changes in consciousness [2,4].

If no clinical improvement is seen after four weeks, discontinuation is generally advised [3]. If a patient shows improvement, treatment can be continued, with the dose slowly reduced based on clinical recovery and tolerability [1,3]. For older adults, it is important to increase the dose carefully and closely monitor kidney function, since the drug is cleared renally and can accumulate in the setting of renal impairment [3]. Adverse effects such as hallucinations, confusion, or agitation are more common in the elderly, and dose adjustments or discontinuation may be necessary if these occur [3,4].

In Nontraumatic ICU Brain Injury

Although these data are derived from traumatic etiologies, the drug's pharmacologic rationale has led to its off-label use in other acquired nontraumatic brain injuries, including ischemic stroke, intracerebral hemorrhage, subarachnoid hemorrhage, and post-cardiac arrest hypoxic injury, particularly in the intensive care unit (ICU) for patients with persistent disorders of consciousness after sedation weaning [1,7]. Evidence from observational studies supports a potential benefit: in a pooled individual patient data analysis from five cohorts (n = 294) with nontraumatic brain injury, amantadine use was associated with a significantly higher rate of consciousness recovery within five days, though with an increased incidence of epileptic seizures [5].

A 2024 large multicenter retrospective cohort of 442 ICU patients with severe nontraumatic brain injury, in which patients were treated with amantadine between January 2016 and June 2021, reported that 60% of those receiving intravenous amantadine met response criteria (≥3-point GCS increase within five days), with responders showing lower in-hospital mortality and improved functional outcomes at follow-up [6].

Post-stroke and Beyond ICU

In post-stroke rehabilitation, smaller prospective series and systematic reviews (Neurocritical Care 2020) have suggested improvement in arousal, attention, and therapy participation, though high-quality randomized controlled trials remain lacking [1,7]. The ongoing Amantadine for NeuroenhaNcement in acutE patients Study (ANNES trial) is expected to clarify its role in ischemic stroke recovery [8].

In ICU settings, amantadine has been explored as a neurostimulant in patients with prolonged unresponsiveness due to various causes, including stroke. Several observational studies, systematic reviews (Neurocritical Care 2020), and limited randomized trials suggest benefits in improving recovery after stroke, although the evidence is stronger for TBI than non-TBI etiologies [1,7,9]. Amantadine generally has a favorable safety profile, though caution is warranted in elderly patients or those with renal impairment, given the risks of hallucinations or agitation [7,9,10].

The use of amantadine in our patient demonstrates its possible role not only in post-TBI states but also in post-stroke encephalopathy [1,3,7].

Clinicians should consider amantadine in cases where cognitive recovery is delayed or has plateaued once other reversible causes of DoC have been ruled out [1,11]. Early initiation in the ICU or step-down setting may enhance earlier recovery, reduce complications, and improve rehabilitation potential [5-7]. For arousal and consciousness, improvement is often seen within three to seven days in ICU cohorts, whereas in controlled TBI RCTs, functional gains are typically observed over two to four weeks. If no clear benefit is seen by approximately two to four weeks, many teams taper or discontinue the medication [2,5,6,11].

The American Academy of Neurology (AAN) classified amantadine as level B evidence in its recent guidelines for disorders of consciousness [3,11].

Common side effects include insomnia, agitation, and anxiety. Seizure signals were present in some observational studies [10]. Cardiac arrhythmias have been reported in ICU cohorts [6]. The dose should be reduced in CKD, and creatinine should be monitored [12].

Amantadine Versus Other Stimulants

While amantadine has the strongest evidence base for use in nontraumatic disorders of consciousness, other stimulants have been tried, though the supporting data are more limited (Table 1) [1,5,6]. Methylphenidate, a dopamine and norepinephrine reuptake inhibitor, has been shown to improve attention and processing speed in some patients, based on a meta-analysis of randomized controlled trials, especially with longer drug use, but much of its use in this setting is based on the traumatic brain injury literature [13-15]. Modafinil, which promotes wakefulness through hypothalamic orexin activation, has been tested in post-stroke fatigue and hypoarousal states, with mixed results and a typically slower onset of action [16,17]. Bromocriptine is a dopamine receptor agonist, with mixed results, although one study showed it may alter long-term recovery [18,19]. The dopamine precursor combination levodopa/carbidopa has, in some instances, led to an improvement in the level of consciousness in post-stroke cases, resulting in dramatic recoveries [19].

**Table 1:** Comparison of amantadine vs other stimulants for nontraumatic DoC TBI: traumatic brain injury, NMDA: N-methyl-D-aspartate, BID: twice daily, BP: blood pressure, HR: heart rate, D2: dopamine receptor type 2, TID: three times daily, DoC: disorders of consciousness.

Pharmacological Drug	Mechanism of Action	Evidence in Non-TBI	Onset and Duration of Trial	Dose	Advantages	Limitations
Amantadine	Dopaminergic agonist; NMDA receptor antagonist	Moderate - Small Trials and case series support its use [12]	2-4 weeks (response often seen within 1-2 weeks) [2]	100 mg BID[2]	Best evidence among neurostimulants; relatively well tolerated[12]	May cause agitation and hallucinations in the elderly; renal dose adjustment needed [12]
Methylphenidate	Blocks reuptake of dopamine and norepinephrine	Limited - Mostly TBI studies; non-TBI evidence is anecdotal[13-15]	2-14 days initial trials [13-15]	0.3 mg/kg BID[13-15]	Rapid onset; useful in attention or processing speed[13,15]	May increase BP/HR; may carry risk of gastrointestinal upset, agitation, or insomnia[14,15]
Modafinil	Promotes wakefulness via hypothalamic orexin and dopaminergic activation	Sparse data in non-TBI; some post-stroke fatigue studies[16,17]	May take up to 6 weeks[16,17]	100-200 mg Daily [16,17]	May improve alertness and fatigue in hypoactive patients[16,17]	May cause headaches or anxiety; expensive [16,17]
Bromocriptine	Dopamine D2 agonist	Rarely used in non-TBI; isolated reports[18]	Variable [18]	1.25-5 mg BID[18]	Dopaminergic effect like amantadine[18]	Hypotension may occur, nausea[18]
Levodopa/Carbidopa	Dopamine precursor	Case reports in stroke-related akinetic mutism [19]	Days to weeks [19]	Variable (100/25 mg TID)[19]	Strong dopaminergic effect; occasionally dramatic response[19]	Nausea, vomiting, and dizziness may occur[19]

While this case does not provide conclusive evidence, it is consistent with emerging interest in the extended use of amantadine. It calls for expanded clinical trials to assess outcomes in a variety of populations and to explore optimal dosage regimens, duration, and predictors of response. 

## Conclusions

Amantadine may offer a useful treatment option in certain cases of nontraumatic DoC, such as post-stroke encephalopathy and metabolic encephalopathy. In our patient, the remarkable improvement after initiation of amantadine underscores its promise. This report also points to the possible advantages of using amantadine in ICU settings, where starting treatment early for disorders of consciousness could have a substantial effect on recovery. However, more research is still needed to better define its role and determine which patients are most likely to benefit.
